# MGMT and CALCA promoter methylation are associated with poor prognosis in testicular germ cell tumor patients

**DOI:** 10.18632/oncotarget.11167

**Published:** 2016-08-10

**Authors:** Camila Maria da Silva Martinelli, André van Helvoort Lengert, Flavio Mavignier Cárcano, Eduardo Caetano Albino Silva, Mariana Brait, Luiz Fernando Lopes, Daniel Onofre Vidal

**Affiliations:** ^1^ Pediatric Oncology Laboratory, Molecular Oncology Research Center, Barretos Cancer Hospital, Barretos, SP, Brazil; ^2^ Department of Clinical Oncology, Barretos Cancer Hospital, Barretos, SP, Brazil; ^3^ Barretos School of Health Sciences, Dr. Paulo Prata/FACISB, Barretos, SP, Brazil; ^4^ Department of Pathology, Barretos Cancer Hospital, Barretos, SP, Brazil; ^5^ Department of Otolaryngology and Head & Neck Surgery, Johns Hopkins University School of Medicine, Baltimore, MD, USA; ^6^ Barretos Children's Cancer Hospital, Barretos, SP, Brazil; ^7^ Brazilian Childhood Germ Cell Tumor Study Group, Brazilian Pediatric Oncology Society, São Paulo, SP, Brazil

**Keywords:** DNA methylation, biomarkers, prognosis, refractory disease, testicular germ cell tumor

## Abstract

Testicular germ cell tumors (TGCT) represent the second main cause of cancer-related death in young men. Despite high cure rates, refractory disease results in poor prognosis. Epigenetic reprogramming occurs during the development of seminomas and non-seminomas. Understanding the molecular and genetic basis of these tumors would represent an important advance in the search for new TGCT molecular markers. Hence the frequency of methylation of a gene panel (*VGF, MGMT, ADAMTS1*, *CALCA*, *HOXA9, CDKN2B, CDO1* and *NANOG*) was evaluated in 72 primary TGCT by quantitative methylation specific PCR. A high frequency of *MGMT* (90.9%, 20/22; p=0.019) and *CALCA* (90.5%, 19/21; p<0.026) methylation was associated with non-seminomatous tumors while CALCA methylation was also associated with refractory disease (47.4%, 09/19; p=0.005). Moreover, promoter methylation of both genes predicts poor clinical outcome for TGCT patients (5-year EFS: 50.5% vs 77.1%; p=0.032 for *MGMT* and 51.3% vs 77.0%; p=0.029 for *CALCA*). The findings of this study indicate that methylation of *MGMT* and *CALCA* are frequent and could be used as new molecular markers of prognosis in TGCT.

## INTRODUCTION

Testicular germ cell tumors (TGCT) comprise around 98% of all testicular neoplasms, being the most common malignant neoplasm in young men between 20 and 35 years of age. Approximately, 3 to 6 new cases of TGCT are diagnosed per 100,000 men/year in industrialized countries, representing the second main cause of cancer-related death in this age group [1]. TGCT are classified in two major histological groups, seminomas (SE) and non-seminomas (non-SE) that frequently occur as mixed tumors presenting several histologies [2]. In the last decades, TGCT treatment has been based on platinum chemotherapy and surgery, resulting in high cure rates [3–5]. However, approximately 15% of the patients will present refractory disease resulting in very poor prognosis [6]. Currently, the risk stratification of the patients is based exclusively on clinical parameters and serum markers [7].

Strong evidences suggest that the first event in the pathogenesis of TGCT occurs during the embryonic development. Besides the histological heterogeneity of these tumors, it is proposed that they arise from the same cell, the primordial germ cell, sharing specific gene expression profiles and epigenetic patterns [8]. Once TGCT emerge at a germ cell development time, in which a genome-wide methylation erasure occurs, a failure in the reestablishment of these marks, could lead to wrong epigenetic reprogramming and aberrant growth of these cells, thus contributing to tumor initiation [9, 10].

DNA methylation, the most studied epigenetic mechanism, can inhibit gene transcription, leading to gene silencing [11]. Aberrant methylation of gene promoter regions has been associated with the initiation and progression of several tumors. Genes with tumor suppressive potential such as *BRCA1*, *TP53*, *RASSF1A*, *CALCA* and *MGMT* have been frequently described as methylated in a series of tumors [12]. Several studies evaluated the profile of gene promoter methylation in TGCT and reported significant differences in the pattern of methylation between SEs and non-SEs [13–16].

The biological differences between SEs and non-SEs may reflect the clinical behavior and treatment resistance in TGCT patients. Therefore, the understanding of the DNA methylation profile of these tumors may lead to an important advance in the identification of potential molecular markers for prognosis prediction and/or treatment of TGCT patients.

In this study, 72 primary TGCT samples (of which 20 were SEs and 52 were non-SEs) were evaluated using a candidate gene approach, in order to assess a set of potential biomarkers that would accurately discriminate clinical outcome of patients with TGCT. Four of the genes (*MGMT*, *VGF*, *HOXA9* and *NANOG*) had been previously evaluated for methylation status in TCGT [13–16]. The remaining four genes (*CALCA*, *CDKN2B*, *CDO1* and *ADAMTS1*) have been described to be methylated in other cancer types, but had never been assessed in TGCT.

## RESULTS

### TGCT population characteristics

The mean age of the patients included was 29.6 years (range 26 to 32). It was observed that 72.2% of the cases presented non-seminomas (52/72), being 63.5% (33/52) composed by mixed tumors, and 86.1% (62/72) had advanced disease stage (IS, II or III). In addition, 70.8% (51/72) presented metastasis at diagnosis in the frequently affected sites: lung, liver, bones and/or lymph nodes. Increased levels of serum markers (BHCG, AFP and DHL) were observed in 76.4% (55/72) of the cases. Additionally, 20.8% (15/72) presented refractory disease to standard chemotherapy. According to IGCCCG (*International Germ Cell Cancer Collaborative Group*) [7], 55.5% (40/72) of the cases were classified as good risk disease. The clinicopathological characteristics of the TGCT population are summarized in Table 1.

**Table 1:** Clinicopathological characteristics of the testicular germ cell tumor patients

Clinicopathological characteristics		N	%
**Age (years)**	<29.6	42	58.3
	≥29.6	30	41.7
**Histologic classification**	Seminomas	20	27.8
	Non-seminomas	52	72.2
**Histology**	Seminoma	20	27.8
	Embryonal carcinoma	10	13.9
	Mature teratoma	01	1.4
	Imature Teratoma	03	4.2
	Yolk sac tumor	05	6.9
	Mixed GCT	33	45.8
**Clinical stage (AJCC)**	I	10	13.9
	IS / II / III	62	86.1
**Metastasis (diagnosis)**	No	21	29.2
	Yes	51	70.8
**Serum markers (AJCC)**	S0	05	6.9
	S1	28	38.9
	S2	23	31.9
	S3	04	5.6
	SX	12	16.7
**Refractory disease**	No	47	65.3
	Yes	15	20.8
	No chemotherapy	07	9.7
	Missing	03	4.2
**Chemotherapy regimen^1^**	BEP	50	69.4
	EP	12	16.6
	Carboplatin	01	1.4
	Other	01	1.4
	No chemotherapy	07	9.8
	Missing	01	1.4
**Chemotherapy cycles^2^**	1 cycle	03	4.7
	2 cycles	04	6.3
	3 cycles	19	29.7
	4 cycles	37	57.8
	Missing	01	1.5
**IGCCCG risk**	Good	40	55.5
	Intermediate	13	18.0
	Poor	6	8.3
	Not applicable	13	18.0

**AJCC:** American Joint Committee on Cancer; **IGCCCG:** International Germ Cell Cancer Cooperative Group.

^1^ **BEP:** bleomycin, etoposide and cisplatin; **EP:** Etoposide and cisplatin.

^2^ Based on the information of total 64 patients that received chemotherapeutic treatment.

### Association between the frequency of gene methylation and clinicopathological characteristics

First, a methylation cut-off value was determined to discriminate patients with good outcome (without occurrence of events) from patients with poor outcome (with occurrence of any event during the follow-up) using ROC curve analysis (Supplementary Table S1). Gene methylation frequency was determined based on the establishment of the cut-off value (Supplementary Table S2).

A significant association of *CALCA* methylation with histological classification was observed, in which 90.5% (19/21) of methylated cases were classified as non-SEs (p=0.026). A higher frequency of *CALCA* methylated samples in embryonal carcinomas or yolk sac tumors (p=0.017) was also observed. Furthermore, a significant association was found regarding refractory disease, in which patients that presented refractory disease were frequently methylated (47.4% (09/19); compared to 14.0% (06/43) of patients with unmethylated promoter status) (p=0.005; Table 2).

**Table 2:** Association of the frequency of *CALCA* methylation with clinicopathological characteristics of the patients

Clinicopathological characteristics		*CALCA*		p-value
		UnmethylatedN (%)	MethylatedN (%)	
**Age (years)**	<29.6	28 (54.9)	14 (66.7)	0.357
	≥29.6	23 (45.1)	07 (33.3)	
**Histologic classification**	Seminoma	18 (35.3)	02 (9.5)	**0.026**
	Non-seminoma	33 (64.7)	19 (90.5)	
**Histology**	Seminoma	18 (35.3)	02 (9.5)	**0.017**
	Embryonal carcinoma	05 (9.8)	05 (23.8)	
	Mature teratoma	01 (2.0)	0 (0.0)	
	Imature teratoma	02 (3.9)	01 (4.8)	
	Yolk sac tumor	01 (2.0)	04 (19.0)	
	Mixed GCT	24 (47.1)	09 (42.9)	
**Clinical stage**	I	09 (17.6)	01 (4.8)	0.151
	IS / II / III	42 (82.4)	20 (95.2)	
**Metastasis (diagnosis)**	No	17 (33.3)	04 (19.0)	0.225
	Yes	34 (66.7)	17 (81.0)	
**Serum markers**	S0	05 (11.6)	0 (0.0)	0.399
	S1	20 (46.5)	08 (47.1)	
	S2	16 (37.2)	07 (41.2)	
	S3	02 (4.7)	02 (11.8)	
**Refractory disease**	No	37 (86.0)	10 (52.6)	**0.005**
	Yes	06 (14.0)	09 (47.4)	
**IGCCCG risk**	Good	32 (76.2)	08 (47.1)	0.057
	Intermediate	07 (16.7)	06 (35.3)	
	Poor	03 (7.1)	03 (17.6)	

Bold p-value: statistically significant; p<0.05.

Multiple logistic regression analysis revealed that *CALCA* methylation is significantly associated with non-SE histology and refractory disease, independently of any other characteristic. Patients with non-SE tumors and patients with refractory disease presented a 9.94 and 4.64 times, respectively, greater chance to present *CALCA* promoter methylation (p=0.036 and p=0.023, respectively) (Supplementary Table S3).

Similarly, a significant association of *MGMT* methylation frequency with the histological classification of the tumors was observed, in which 90.9% (20/22) of methylated cases were classified as non-seminomas (p=0.019). A marginal significance value related to refractory disease was also observed, in which 38.1% (08/21) of the tumors presenting *MGMT* methylation were refractory to standard platinum-based chemotherapy (p=0.067, Table 3).

**Table 3:** Association of the frequency of *MGMT* methylation with clinicopathological characteristics of the patients

Clinicopathological characteristics		*MGMT*		p-value
		UnmethylatedN (%)	MethylatedN (%)	
**Age (years)**	<29.6	27 (54.0)	15 (68.2)	0.261
	≥29.6	23 (46.0)	07 (31.8)	
**Histologic classification**	Seminoma	18 (36.0)	02 (9.1)	**0.019**
	Non-seminoma	32 (64.0)	20 (90.9)	
**Histology**	Seminoma	18 (36.0)	02 (9.1)	0.115
	Embryonal carcinoma	06 (12.0)	04 (18.2)	
	Mature teratoma	01 (2.0)	0 (0.0)	
	Imature teratoma	01 (2.0)	02 (9.1)	
	Yolk sac tumor	03 (6.0)	02 (9.1)	
	Mixed GCT	21 (42.0)	12 (54.5)	
**Clinical stage**	I	08 (16.0)	02 (9.1)	0.713
	IS / II / III	42 (84.0)	20 (90.9)	
**Metastasis (diagnosis)**	No	14 (28.0)	7 (31.8)	0.743
	Yes	36 (72.0)	15 (68.2)	
**Serum markers**	S0	03 (7.3)	02 (10.5)	0.672
	S1	21 (51.2)	07 (36.8)	
	S2	15 (36.6)	08 (42.1)	
	S3	02 (4.9)	02 (10.5)	
**Refractory disease**	No	34 (82.9)	13 (61.9)	0.067
	Yes	07 (17.1)	08 (38.1)	
**IGCCCG risk**	Good	30 (73.2)	10 (55.6)	0.134
	Intermediate	09 (22.0)	04 (22.2)	
	Poor	02 (4.9)	04 (22.2)	

Bold p-value: statistically significant; p<0.05.

Multiple logistic regression analysis revealed that *MGMT* methylation is significantly associated with non-SE histology, independently of any other characteristic. Patients with non-SE tumors had 5.6 times higher chance to present *MGMT* methylation (p=0.03, CI: 1.177 – 26.877) (Supplementary Table S4).

Interestingly, an association between *MGMT* and *CALCA* methylation in TGCT was observed, in which for patients with *MGMT* methylation, 59.1% (13/22) also presented *CALCA* methylation, while in the *MGMT* unmethylated patients group, only 16.0% (8/50) presented *CALCA* methylation (p<0.001; Chi-square test).

For *VGF, NANOG*, *CDKN2B, CDO1* and *ADAMTS1*, no statistical associations with clinicopathological characteristics were observed (data not shown). For *HOXA9*, it was observed that all methylated samples were non-SEs (14/52), and SEs (0/20) showed no methylation for this gene (p<0.01).

### Association of the frequency of *MGMT* and *CALCA* methylation with prognosis of TGCT patients

**Figure 1:**
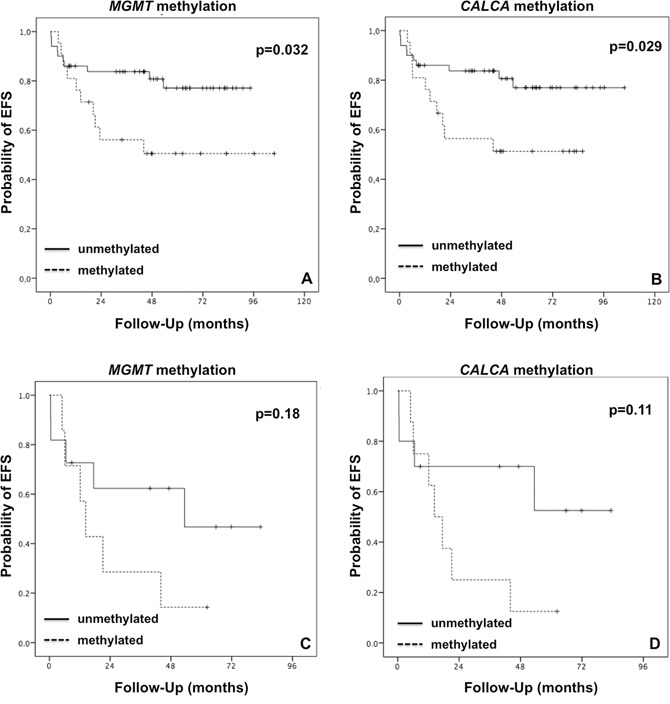
Kaplan-Meier survival curves for event-free survival (EFS) regarding methylation of *MGMT* or *CALCA* in the TCGT patients **A-B.** all TGCT patients; **C-D.** IGCCCG intermediate and poor risk TGCT patients. The differences between the survival curves were analyzed using the Log-rank test. Statistical significance was considered for p<0.05.

A 5-year overall survival (OS) of 83.1% and 5-year event-free survival (EFS) of 68.7% for the population of this study was observed. Regarding EFS, only one death due to toxicity and 10 deaths from disease were observed. No significant difference was observed in OS rates according to the *MGMT* or *CALCA* methylation frequency independently (p=0.386 and p=0.192, respectively; Supplementary Table S5). However, in patients that presented *MGMT* promoter methylation compared to patients with unmethylated promoter status, a significant lower 5-year EFS rate was observed (50.5% vs 77.1%; p=0.032; Figure 1A; Supplementary Table S6). Similarly, for the patients that presented *CALCA* promoter methylation compared to patients with unmethylated promoter status, a significant lower 5-year EFS rate was also observed (51.3% vs 77.0%; p=0.029; Figure 1B; Supplementary Table S6).

Additionally, the data obtained from this study revealed that alterations in serum markers, refractory disease and intermediate/poor IGCCCG risk are significantly associated with decreased OS rates of the patients (Supplementary Table S5). Regarding EFS, the results revealed that histology, alterations in serum markers, metastasis at diagnosis, refractory disease and intermediate/poor IGCCCG risk are significantly associated with decreased 5-year EFS rates (Supplementary Table S6). COX regression analysis for OS and EFS demonstrated that IGCCCG risk emerged as the unique independent prognostic parameter in TGCT (Supplementary Tables S7 and S8).

Approximately, 20% of patients classified with intermediate/poor IGCCCG risk presented refractory disease and/or relapse in TGCT. The prognostic role of *MGMT* and *CALCA* methylation status in these patients was investigated (13 intermediate and 6 poor). Despite the fact that EFS analysis did not show a significant difference regarding *MGMT* methylation profile, patients with *MGMT* promoter methylation compared to patients with unmethylated promoter status presented lower 5-year EFS (14.3% vs 46.8%; p=0.18; Figure 1C). The same result was observed for *CALCA*; patients with *CALCA* promoter methylation compared to patients with unmethylated promoter status presented lower 5-year EFS (12.5% vs 52.5%; p=0.11; Figure 1D).

## DISCUSSION

In this study, the frequency of promoter methylation of eight candidate genes (*MGMT, CALCA*, *VGF, HOXA9, NANOG*, *CDKN2B, CDO1* and *ADAMTS1*) was evaluated to assess a set of potential biomarkers that would accurately discriminate clinical outcome of patients with TGCT. A significant proportion of TGCT patients (15-25%) presents refractory disease, which results in a poor outcome [17, 18]. Therefore, efforts to identify new molecular prognostic markers are essential in TGCT, especially for these refractory patients [19].

In this study, a high frequency of *CALCA* and *MGMT* methylation was found to be associated with non-SE tumors and poor clinical outcome in TGCT patients, through promoter methylation profiling analysis using a candidate gene approach by QMSP.

The role of *CALCA* methylation in human cancer is not clear. *CALCA* is a potent vasodilator and also acts in bone metabolism through the regulation of calcium [20]. Frequent methylation of *CALCA* has been reported in hematological cancers, especially in myelodysplastic syndromes [21] and in acute leukemia, in which it was associated with a higher risk of relapse and poor clinical outcome [22, 23]. *CALCA* methylation has also been described in several types of solid tumors [24–26] and it was associated with poor clinical outcome of patients with non-small cell lung cancer [25]. To our knowledge, this is the first report demonstrating that *CALCA* methylation is associated with non-SE tumors, refractory disease and poor clinical outcome in TGCT.

*MGMT* methylation has been extensively reported in TGCT and was associated with non-SE tumors [16, 27, 28]. This study observed a higher frequency of *MGMT* methylation associated with non-SE tumors than other reports (90% vs 50-69%) [16, 27]. This difference is possibly due to the approach used in the present study of setting the methylation level cut-off based on the discrimination of prognosis instead in normal versus tumor tissues, as performed in those previous studies. However, our data are consistent with the observation of a high frequency of *MGMT* methylation in non-SEs.

We also observed that *MGMT* methylation is associated with a worse clinical outcome in TGCT patients (p=0.032; Figure 1A) and tends to be more frequent in patients with refractory disease (p=0.067, Table 2). *MGMT* encodes the O-6-methylguanine-DNA methyltransferase protein involved in DNA repair by removing the alkyl groups of O^6^-methylguanine and preventing DNA lesions. These alkyl groups mainly occur as a result of the use of alkylating agents, such as temozolomide [29]. In TGCT, the treatment is based on the use of cisplatin combined with etoposide and bleomycin, and none of these drugs are alkylating agents. The role of *MGMT* methylation in cisplatin-resistance in TGCT is not clear. Deficient DNA repair mechanism attributed to *MGMT* disruption does not seems to be involved in the mechanisms of cisplatin-resistance in TGCT [30].

The observation that *MGMT* methylation is more frequent in non-SEs, a subtype that usually presents a more aggressive behavior suggests that alkylating agents (i.e. temozolomide), could be used in the treatment of TGCT. Cell lines derived from human non-SEs TGCT showed sensitivity to temozolomide, however, the status of *MGMT* methylation was not evaluated [31]. Results reported from two phase II clinical trials were unfavorable regarding the use of temozolomide in patients diagnosed with relapsed or cisplatin-refractory germ cell tumors. One study showed no effect of temozolomide in the treatment of cisplatin-refractory GCT patients [32], while the other demonstrated that although well tolerated, only 10% (2/20) of the cisplatin-refractory GCT patients achieved partial responses after temozolomide treatment [33]. However, the use of temozolomide in these trials was not based on the *MGMT* methylation status of the patients, which in part can explain these dismal findings.

Considering the higher methylation levels found in refractory disease and poor clinical outcome in TGCT patients, we also argue that these groups could possibly benefit from a therapy based on DNA methylation inhibitors (i.e 5-aza-CdR) as a single or combined agent. There is currently little evidence available on the effects of DNA methylation inhibitors in TGCT, but it has been shown that TGCT cells may be markedly sensitive to DNA methylation inhibitors and that a pretreatment using 5-aza-CdR can restore cisplatin cytotoxicity to resistant embryonal carcinoma [34].

It is well known that IGCCCG risk is the most important parameter to establish prognosis in TGCT [7]. However, this parameter is not sufficient to predict which patients will present refractory disease, consequently evolving with disease progression or relapse. Regarding IGCCCG risk, the group of patients classified as intermediate/poor risk is of particular interest, because these are the patients that present refractory disease and/or relapse [7]. In our study, 19 patients were classified in these groups and despite the fact that there was no statistical significance, data from this study demonstrated that intermediate/poor risk patients methylated for *MGMT* or *CALCA* presented lower 5-year EFS rates. *MGMT* and *CALCA* promoter methylation analysis should be performed in a large number of intermediate/poor risk TGCT patients to confirm these findings.

In conclusion, our data demonstrated that *CALCA* and *MGMT* are frequently methylated in non-SEs and are associated with poor clinical outcomes in TGCT patients. Additionally, our results indicate that *CALCA* methylation is associated with refractory disease. Therefore, our data support the use of *CALCA* and *MGMT* methylation status as new molecular prognostic markers in TGCT. However, their predictive role for the use of new treatment strategies (i.e. temozolomide or DNA methylation inhibitors) in TGCT should be further explored in prospective clinical studies.

## MATERIALS AND METHODS

### Samples

Formalin-fixed paraffin-embedded (FFPE) tissues from 72 cases of primary testicular germ cell tumors (SE, non-SE and mixed tumors), diagnosed between 2005 and 2012, were retrieved from the archives of the Department of Pathology at the Barretos Cancer Hospital. The samples were from primary tumors, collected prior to the use of any systemic treatment. For each case, a slide stained with hematoxylin and eosin (H&E) was analyzed by a pathologist (ECAS) to confirm the diagnosis and to determine areas with more than 70% of tumor cellularity for DNA extraction. Refractory disease was defined as failure in decrease of serum marker levels after the first line chemotherapy or early relapse (relapse within 2 years from the first therapeutic approach).

The study was conducted following the national and institutional ethical policies and was approved by the Barretos Cancer Hospital Ethical Committee (protocol CAAE 26848814.8.0000.5437).

### DNA extraction and bisulfite treatment

DNA was isolated from four FFPE tissue sections of 10μm using standard DNA Mini kit protocol (Qiagen). Sodium bisulfite treatment of 2μg genomic DNA was performed with EZ DNA Methylation Kit (Zymo Research) according to manufacturer's recommendations. The bisulfite-modified genomic DNA was eluted in 40μL of ultra-pure water (milliQ) and stored at -80°C.

The conversion efficiency of bisulfite treatment was verified through the direct sequencing of a PCR product generated with *ACTB* primers designed for the analysis of converted DNA sequences (Supplementary Table S9) in 12 randomly selected samples after bisulfite conversion, to exclude bias due to incomplete bisulfite conversion. Briefly, PCR was performed using 50ng of converted DNA, 1X enzyme buffer, 0.2mM dNTPs, 2mM MgCl_2_, 0.3μM of each *ACTB* primers and 0.5U of Platinum Taq DNA Polymerase (Invitrogen), in a final volume of 25µL following the conditions: 95°C for 10 min, followed by 40 cycles at 95°C for 30 seconds, 56°C for 30 seconds and 72°C for 30 seconds, and 72°C for 10 minutes. The reaction was carried out in a Veriti Thermal Cycler (Applied Biosystems) and PCR product was checked in a 1% agarose gel. The fragment with expected size (133bp) was purified from the agarose gel using Wizard SV Gel and PCR Clean-up System (Promega) and direct sequenced using the standard DNA Sequencing Big Dye Terminator v3.1 kit (Applied Biosystems). The sequencing reaction was cleaned-up with Big Dye X Terminator purification kit (Applied Biosystems) and the plate was directly loaded onto an Applied Biosystems 3500 genetic analyzer. All unmethylated cytosines in *ACTB* amplicon were completely converted to thymines, confirming the high efficiency of the of bisulfite treatment (Supplementary Figure S1).

### Methylation analysis

Bisulfite-modified DNA was used as a template for the quantitative methylation specific PCR (QMSP), as previously described [16].

Briefly, amplification reactions were performed in a final volume of 20μL in the following conditions: 1μL (~50ng) of genomic bisulfite-modified DNA; 600nM of forward and reverse primers, 200nM probe, 0.6U of Platinum Taq DNA polymerase (Invitrogen), 200μM dNTPs, 6.7mM MgCl_2_ and 1X ROX Reference Dye (BioRad). Primers and probes were used to specifically amplify each gene promoter region and the promoter region of *ACTB*, in an area without CG nucleotides, so it could be used as a reference gene. For six genes (*MGMT, CALCA*, *VGF, HOXA9, CDKN2B* and *CDO1*) primers and probes sequences were selected from the literature [16, 35–38]. For *NANOG* and *ADAMTS1*, primers and probes were designed following recommendations from Eads et al. (2000) [35]. The sequences of primers and probes are described in Supplementary Table S9.

The reactions were performed following the conditions: 95°C for 3 minutes, followed by 50 cycles at 95°C during 15 seconds and 60°C during 1 minute. Reactions were carried out in 96-well plates in a 7900HT Fast Real-Time PCR system (Applied Biosystems) and results were analyzed using the software SDS2.4 (Applied Biosystems). The reactions were performed in triplicates and each run included a positive (*in vitro* methylated normal leukocyte DNA) and negative (normal leukocyte DNA) controls and multiples no template controls. Leukocyte DNA from a healthy individual was submitted to *in vitro* treatment with *SssI* methyltransferase (New England Biolabs) to generate a virtually completely methylated DNA. It was used as a template for the design of the standard curve through serial dilutions (50-0.005ng) in each plate. The relative level of methylation for each gene in each sample was considered as the ratio of the triplicates’ average of the gene of interest to the triplicates’ average of *ACTB*, multiplied by 1000 for easier tabulation. The samples were considered methylated when at least two of the triplicates were amplified. The reaction was repeated in the cases with amplification of only one of the triplicates and considered positive if amplification was confirmed.

### Statistical analysis

To categorize gene methylation frequency, Receiver-operating characteristics (ROC) analysis was used to determine a cut-off that maximized the sensitivity and specificity to discriminate the outcome of the patients. For this analysis, the terms good or poor clinical outcome was assigned for those patients without or with the occurrence of events during the follow-up, respectively. Area under the curve (AUC), sensitivity, specificity, positive predictive value, negative predictive value, accuracy and 95% confidence intervals were estimated. Furthermore, the association of gene methylation frequency with clinicopathological characteristics was evaluated using Chi-square or Fisher's exact test. Subsequently, multiple logistic regression was performed, considering the variables with p<0.2 in the previous test.

Overall survival (OS) and event-free survival (EFS) were assessed by Kaplan-Meier method. For OS, event was considered as death from any cause and the follow-up from the date of diagnosis to the date of death. For EFS, event was considered as progression, recurrence or death and follow-up from the date of diagnosis to the date of the event that occurred first. The differences between the survival curves were analyzed using the Log-rank test. Subsequently, COX regression was performed considering the variables with p<0.2 in the survival analysis.

For all analyzes, p<0.05 was considered for statistical significance. Statistical analysis was developed using SPSS v.21 software.

## SUPPLEMENTARY MATERIALS FIGURE AND TABLES


