# Supplementary material for: MGMT and CALCA promoter methylation are associated with poor prognosis in testicular germ cell tumor patients
Source: Oncotarget. 2016 Aug 10;8(31):50608–17. doi: 10.18632/oncotarget.11167 (PMC5584175; doi:10.18632/oncotarget.11167)
Supplement: Supplementary file 1 [file oncotarget-08-50608-s001.pdf]

MGMT and CALCA promoter methylation are associated with poor prognosis in testicular germ cell tumor patients

SUPPLEMENTARY MATERIALS

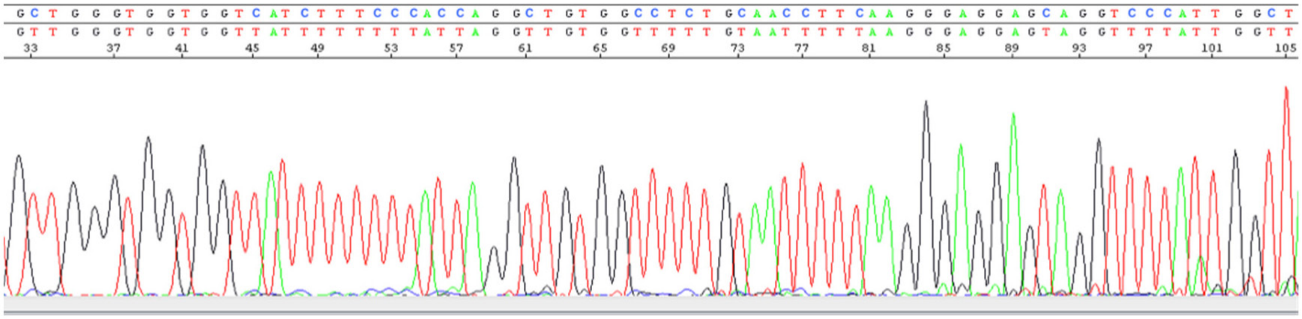

Supplementary Figure S1: Representative electropherogram of an amplified fragment of *ACTB* promoter region. Sequence above represents the unconverted genomic DNA and indicates the original positions of cytosines that were completely converted to thymines after bisulfite modification (sequence below). *ACTB* primers were designed for the amplification of the bisulfite-converted DNA sequence (Supplementary Table S9).

Supplementary Table S1: Methylation cut-off based on predictive probability of clinical outcome among tumor patients

Gene	Cut-off*	Sensitivity (%)	Specificity (%)	PPV	NPV	Accuracy
VGF	1.81	25.0 (8.7 – 49.1)	90.4 (79.0 – 96.8)	50.0 (18.9 – 81.1)	75.8 (63.3 – 85.8)	72.2 (61.0 – 81.2)
MGMT	3.70	50.0 (27.2 – 72.8)	76,9 (63.2 – 87.5)	45.5 (24.4 – 67.8)	80.0 (66.3 – 90.0)	69.4 (58.1 – 79.0)
ADAMTS1	6.15	10.0 (1.5 – 31.7)	98.1 (89.7 – 99.7)	66.7 (11.6 – 94.5)	73.9 (61.9 – 83.7)	73.6 (62.4 – 82.4)
CDO1	145.66	15.0 (3.4 – 37.9)	98.1 (89.7 – 99.7)	75.0 (20.3 – 95.9)	75.0 (63.0 – 84.7)	75.0 (63.9 – 83.6)
HOXA9	14.64	20.0 (5.9 – 43.7)	80.8 (67.5 – 90.4)	28.6 (8.6 – 58.1)	72.4 (59.1 – 83.3)	63.9 (52.4 – 74.0)
CALCA	84.28	50.0 (27.2 – 72.8)	78.9 (65.3 – 88.9)	47.6 (25.7 – 70.2)	80.4 (66.9 – 90.2)	70.8 (59.5 – 80.1)
CDKN2B	33.00	65.0 (40.8 – 84.5)	57.7 (43.2 – 71.3)	37.1 (21.5 – 55.1)	81.1 (64.8 – 92.0)	59.7 (48.2 – 70.3)
NANOG	34.56	52.9 (27.9 – 77.0)	63.8 (48.5 – 77.3)	34.6 (17.3 – 55.7)	79.0 (62.7 – 90.4)	54.2 (42.7 – 65.2)

*The optimal cutoff was determined as the point at which it simultaneously maximized sensitivity and specificity; **PPV:** positive predictive value; **NPV:** negative predictive value.

Supplementary Table S2: Gene promoter methylation frequency according to clinical outcome of TGCT patients

		Outcome*	
		Good	Poor
Gene	Cut-off	Number of tumors with methylation/ total number of tumors (%, 95% CI)	Number of tumors with methylation/ total number of tumors (%, 95% CI)
VGF	1.81	05 / 52 (9.6, 6.0 – 12.37)	05 / 20 (25.0, 0 – 159.1)
MGMT	3.70	12 / 52 (23.1, 0 – 96.66)	10 / 20 (50.0, 0 – 785.1)
ADAMTS1	6.15	01 / 52 (1.9, 0 – 9.4)	02 / 20 (10.0, 0 – 52.0)
CDO1	145.66	01 / 52 (1.9, 0 – 16.9)	03 / 20 (15.0, 0 – 327.9)
HOXA9	14.64	10 / 52 (19.3, 9.42 – 68.9)	04 / 20 (20.0, 0 – 111.7)
CALCA	84.28	11 / 52 (21.1, 35.42 – 166.8)	10 / 20 (50.0, 0 – 2255,4)
CDKN2B	33.00	30 / 52 (57.7, 34.5 – 60.4)	07 / 20 (35.0, 18.4 – 41.9)
NANOG	34.56	17 / 47 (36.2, 206.1 – 1020.5)	09 / 17 (53.0, 0 – 2042.9)

*The terms good or poor clinical outcome were assigned for those patients without or with the occurrence of events during the follow-up, respectively. Event was considered as progression, recurrence or death.

Supplementary Table S3: Multiple logistic regression for *CALCA* promoter methylation and clinicopathological characteristics of TGCT patients

Variable	Category	Odds Ratio	95% CI (for Exp β)	p-value
Histologic Classification	Seminoma	1	-	-
	Non-seminoma	9.936	1.163 – 84.867	**0.036**
Refractory disease	No	1	-	-
	Yes	4.639	1.236 – 17.409	**0.023**
Constant		0.043	-	0.003

Bold: significant p-value <0.05.

Supplementary Table S4: Multiple logistic regression for *MGMT* promoter methylation and clinicopathological characteristics of TGCT patients

Variable	Category	Odds Ratio	95% CI (for Exp β)	p-value
Histologic Classification	Seminoma	1	-	-
	Non-seminoma	5.625	1.177 – 26.877	**0.03**
Constant		0.111	-	0.003

Bold: significant p-value <0.05.

Supplementary Table S5: Five-year overall survival according to clinicopathological and molecular characteristics of TGCT patients

Variables	Category	Cases (N)	Events (N)	Overall survival probability (%, 5 years)	p-value
Age (years)	< 29.6	41	05	86.4	0.129
	> 29.6	30	08	78.3	
Histologic classification	Seminomas	20	04	78.5	0.532
	Non-seminomas	51	09	85.1	
Histology	Seminoma	20	04	78.5	0.061
	Embryonal carcinoma	10	0	100.0	
	Mature teratoma	01	0	100.0	
	Immature teratoma	03	01	66.7*	
	Yolk sac tumor	05	03	60.0	
	Mixed GCT	32	05	90.6	
Clinical stage	I	10	0	100.0	0.129
	IS / II / III	61	13	80.5	
Metastasis (diagnosis)	No	21	01	100.0	0.061
	Yes	50	12	76.6	
Serum markers	S0	05	0	100.0	**0.032**
	S1	28	03	88.8	
	S2	22	06	76.3	
	S3	04	02	50.0*	
Refractory disease	No	46	03	93.1	**0.003**
	Yes	15	06	68.5	
IGCCCG risk	Good	40	03	94.7	**0.003**
	Intermediate	13	05	66.1	
	Poor	05	02	60.0*	
VGF	Unmethylated	61	10	86.1	0.224
	Methylated	10	03	63.0	
MGMT	Unmethylated	50	07	85.6	0.386
	Methylated	21	06	77.9	
ADAMTS1	Unmethylated	68	12	84.4	0.504
	Methylated	03	01	50.0	
CDO1	Unmethylated	67	11	85.9	0.092
	Methylated	04	02	37.5	
HOXA9	Unmethylated	58	10	85.4	0.470
	Methylated	13	03	74.6	
CALCA	Unmethylated	50	07	87.7	0.192
	Methylated	21	06	73.7	
CDKN2B	Unmethylated	34	09	73.4	0.088
	Methylated	37	04	91.4	
NANOG	Unmethylated	37	06	84.9	0.914
	Methylated	26	05	83.9	

Bold: significant p-value <0.05; *: 3-years overall survival.

Supplementary Table S6: Five-year event-free survival according to clinicopathological and molecular characteristics of TGCT patients

Variables	Category	Cases (N)	Events (N)	Event-free survival probability (%, 5 years)	p-value
Age (years)	< 29.6	41	10	71.6	0.275
	> 29.6	30	10	65.6	
Histologic classification	Seminomas	20	04	78.5	0.436
	Non-seminomas	51	16	65.1	
Histology	Seminoma	20	04	78.5	**0.039**
	Embryonal carcinoma	10	01	85.7	
	Mature teratoma	01	0	100.0	
	Immature teratoma	03	02	33.3*	
	Yolk sac tumor	05	04	20.0	
	Mixed GCT	32	09	69.0	
Clinical stage	I	10	0	100.0	0.056
	IS / II / III	61	20	64.2	
Metastasis (diagnosis)	No	21	01	95.2	**0.009**
	Yes	50	19	58.9	
Serum markers	S0	05	0	100.0	**0.010**
	S1	28	03	88.8	
	S2	22	10	51.5	
	S3	04	04	0	
Refractory disease	No	46	03	93.1	**<0.001**
	Yes	15	13	7.5	
IGCCCG risk	Good	40	06	84.0	**<0.001**
	Intermediate	13	06	46.4.1	
	Poor	05	05	0	
VGF	Unmethylated	61	15	72.6	0.091
	Methylated	10	05	46.7	
MGMT	Unmethylated	50	10	77.1	**0.032**
	Methylated	21	10	50.5	
ADAMTS1	Unmethylated	68	18	70.8	0.211
	Methylated	03	02	33.3	
CDO1	Unmethylated	67	17	72,0	0.059
	Methylated	04	03	25.0	
HOXA9	Unmethylated	58	16	69.3	0.909
	Methylated	13	04	68.4	
CALCA	Unmethylated	50	10	77.0	**0.029**
	Methylated	21	10	51.3	
CDKN2B	Unmethylated	34	13	55.0	0.086
	Methylated	37	07	80.7	
NANOG	Unmethylated	37	08	76.1	0.325
	Methylated	26	09	60.3	

Bold: significant p-value <0.05.

Supplementary Table S7: COX regression model for overall survival considering the clinical, pathological and molecular characteristics of TGCT patients

Variable	Category	Hazard ratio	95% CI (for Exp β)	p-value
Age (years)	< 29.6	1		
	> 29.6	4.039	0.314 – 52.004	0.284
Histologic Classification	Seminoma	1	-	-
	Non-seminoma	0.472	0.053 – 4.176	0.500
Refractory disease	No	1	-	-
	Yes	6.616	0.741 – 59.049	0.091
IGCCCG risk	Good	1		
	Intermediate	20.49	1.223 – 343.268	**0.036**
	Poor	45.771	0.462 – 343.031	0.103
MGMT methylation	Unmethylated	1		
	Methylated	2.316	0.143 – 37.622	0.555
CALCA methylation	Unmethylated	1		
	Methylated	0.116	0.002 – 1.937	0.116

Bold: significant p-value <0.05.

Supplementary Table S8: COX regression model for event-free survival considering the clinical, pathological and molecular characteristics of TGCT patients

Variable	Category	Hazard ratio	95% CI (for Exp β)	p-value
Histologic Classification	Seminoma	1	-	-
	Non-seminoma	0.987	0.253 – 3.853	0.985
IGCCCG risk	Good	1		
	Intermediate	4.031	1.157 – 14.040	**0.029**
	Poor	20.448	3.829 – 109.206	**<0.001**
MGMT methylation	Unmethylated	1		
	Methylated	2.087	0.547 – 7.964	0.282
CALCA methylation	Unmethylated	1		
	Methylated	0.591	0.132 – 2.649	0.492

Bold: significant p-value <0.05.

Supplementary Table S9: Primers and probes used for the evaluation of genes promoter methylation in TGCT samples

Gene	Forward (5' – 3')	Probe (6-FAM-5' – 3'-Iowa Black)	Reverse (5' – 3')	Annealing (°C)	Amplicon (bp)	Ref.
ACTB	TGGTGATGGAGGAG GTTTAGTAAGT	ACCACCACCCAACACACAAT AACAAACACA	AACCAATAAAACCTA CTCCTCCCTTAA	60	133	35
VGF	GGATAGCGTTCGT AGGCG	GCGCCCAAAAACGACGTA AACCTAAATAC	AAAAACCGAATT CCCCACCCCG	60	93	16
MGMT	CGAATATACTA AAACAACCCGCG	AATCCTCGCGATACGC ACCGTTTACG	GTATTTTTTCGG GAGCGAGGC	60	122	16
ADAMTS1	TAGGGTGCGTTATCGG	CGCCCCTCTTCGAC CTCCGC	TCTAAAACGCCT CCGAA	60	129	-
CDO1	CCACAACGA CGAAAATAAAACG	TTAACGGCGCGTTTTA GTCGTTCG	TCGGCGTTTTAGG GATCGCG	60	109	38
HOXA9	AATAAATTTTATCGTA GAGCGGTAC	GCGCCCCCATTAACCG TACGCGT	CATATAACAACTTA ATAACACCGAA	60	226	37
CALCA	GTTTTGGAAGTATGA GGGTGACG	ATTCCGCCAATACA CAACAACCAATAAACG	TTCCCGCCGCTATA AATCG	60	101	36
CDKN2B	AGGAAGGAGAGAGT GCGTCG	TTAACGACACTCTTCCCTTC TTTCCCACG	CGAATAATCCACCGT TAACCG	60	81	36
NANOG	TTGAGACGTAGTT TCGTT	CAAAAATTATAATAAACCGA TATCGCGCCAC	AACTACTCTAAAAACT AAAACGA	60	117	-
